# Influenza A virus elicits peri-vascular adipose tissue inflammation and vascular dysfunction of the aorta in pregnant mice

**DOI:** 10.1371/journal.ppat.1010703

**Published:** 2022-08-05

**Authors:** Osezua Oseghale, Stella Liong, Madison Coward-Smith, Eunice E. To, Jonathan R. Erlich, Raymond Luong, Felicia Liong, Mark Miles, Shaghayegh Norouzi, Cara Martin, Sharon O’Toole, Robert D. Brooks, Steven Bozinovski, Ross Vlahos, John J. O’Leary, Doug A. Brooks, Stavros Selemidis

**Affiliations:** 1 School of Health and Biomedical Sciences, RMIT University, Bundoora, Victoria, Australia; 2 Centre for Innate Immunity and Infectious Disease, Hudson Institute of Medical Research, Monash University, Clayton, Victoria, Australia; 3 Department of Pharmacology, Biomedicine Discovery Institute, Monash University, Clayton, Victoria, Australia; 4 Discipline of Histopathology, School of Medicine, Trinity Translational Medicine Institute (TTMI), Trinity College Dublin, Dublin, Ireland; 5 Sir Patrick Dun’s Laboratory, Central Pathology Laboratory, St James’s Hospital, Dublin, Ireland; 6 Emer Casey Research Laboratory, Molecular Pathology Laboratory, The Coombe Women and Infants University Hospital, Dublin, Ireland; 7 CERVIVA research consortium, Trinity College Dublin, Dublin, Ireland; 8 Clinical and Health Sciences, University of South Australia, Adelaide, Australia; Johns Hopkins Bloomberg School of Public Health, UNITED STATES

## Abstract

Influenza A virus (IAV) infection during pregnancy initiates significant aortic endothelial and vascular smooth muscle dysfunction, with inflammation and T cell activation, but the details of the mechanism are yet to be clearly defined. Here we demonstrate that IAV disseminates preferentially into the perivascular adipose tissue (PVAT) of the aorta in mice. IAV mRNA levels in the PVAT increased at 1–3 days post infection (d.p.i) with the levels being ~4–8 fold higher compared with the vessel wall. IAV infection also increased Ly6C^low^ patrolling monocytes and Ly6C^high^ pro-inflammatory monocytes in the vessel wall at 3 d.p.i., which was then followed by a greater homing of these monocytes into the PVAT at 6 d.p.i. The vascular immune phenotype was characteristic of a “vascular storm”- like response, with increases in neutrophils, pro-inflammatory cytokines and oxidative stress markers in the PVAT and arterial wall, which was associated with an impairment in endothelium-dependent relaxation to acetylcholine. IAV also triggered a PVAT compartmentalised elevation in CD4^+^ and CD8^+^ activated T cells. In conclusion, the PVAT of the aorta is a niche that supports IAV dissemination and a site for perpetuating a profound innate inflammatory and adaptive T cell response. The manifestation of this inflammatory response in the PVAT following IAV infection may be central to the genesis of cardiovascular complications arising during pregnancy.

## Author summary

Influenza A virus (IAV) infection remains a major cause of significant disease during pregnancy. IAV infection in pregnancy results in virus dissemination from the lung to the systemic vasculature, thereby initiating profound vascular inflammation and T cell activation that leads to vascular damage. Currently, the details of the mechanism that facilitates this vascular pathology and the influence of IAV dissemination to the vasculature on the perivascular adipose tissue (PVAT) is not clearly defined. Here, we show that IAV disseminates to the PVAT compartment of the vessel at a much larger rate than the vessel wall. We found that IAV infection increased PVAT inflammation characterised by immune cell infiltration, oxidative stress and pro-inflammatory cytokines. This was accompanied by a preferential immune T cell activation in the PVAT. We also found that this vascular inflammatory burden results in vascular endothelial dysfunction that is characterised by an impairment in endothelium dependent relaxation. Our study provides new insights into how IAV utilises the PVAT to promote the vascular inflammatory pathology that disrupts the vasculature in pregnancy and lead to pregnancy complications.

## Introduction

Pregnancy is a risk factor for the severe illness associated with influenza A virus (IAV) infection. The magnitude of the impact on pregnant women during the 1918, 1957, 1968 and 2009 influenza pandemics is highlighted by significant and disproportionately high mortality rates in pregnant women [1]. Seasonal influenza epidemics are also a risk factor for severe disease, hospitalisation and mortality, with IAV-infected pregnant women being 3 to 4 times more likely to be hospitalised due to acute cardiopulmonary events [2]. Morbidity is also increased with advancing gestation, and influenza-infected pregnant women in their third trimester are 5 times more likely to be hospitalised than in the first and second trimester [3]. Moreover, the risk of IAV-induced respiratory failure is increased in pregnant women with underlying co-morbidities, such as hypertension or cardiovascular disease [4]. Pregnancy is a unique state of immunomodulation, which is thought to occur due to the adaptation of the maternal immune system to prevent the rejection of the semi-allogenic fetus, whilst maintaining its ability to clear pathogens. It is postulated that pregnancy-related immunomodulation impacts on IAV-induced disease progression, with increased IAV replication and reduced antiviral gene expression in peripheral blood mononuclear cells (PBMCs), when isolated from pregnant women in their third trimester [5]. Moreover, pregnancy is associated with significant physiological changes to the cardiovascular system, to meet the oxygen and nutrient demands of the developing fetus. Given that IAV-infected pregnant women in their third trimester are more likely to be hospitalised, an increased demand on the maternal cardiovascular system, particularly as pregnancy progresses, may also play a role in increased IAV morbidity in pregnant women.

How pregnancy-related immunomodulation and changes to the cardiovascular system affect IAV disease progression in pregnant women remains poorly understood. Our recent study showed that IAV infection significantly disrupts the normal functioning of the thoracic aorta [6]. We demonstrated that IAV disseminates from the lung into the thoracic aorta to induce a “vascular storm” characterised by increases in inflammatory Ly6G^+^ neutrophils, endothelial patrolling Ly6C^low^ and pro-inflammatory Ly6C^high^ monocytes, as well as CD4^+^ and CD8^+^ T cells. This inflammation was also associated with elevations in inflammatory cytokine expression and oxidative stress in the vessel. We observed substantial oxidative stress within the PVAT of the aorta. The vascular storm resulted in vascular endothelial and smooth muscle dysfunction, which consisted of an impairment in nitric oxide (NO)-dependent vasorelaxation [6]. Although our study highlighted a novel respiratory-vascular disease axis of pathogenesis driven by IAV in pregnancy, the underlying mechanisms remained largely undefined. Here, we hypothesised that the PVAT, which is a major site of immune activation in other cardiovascular diseases such as atherosclerosis [7,8] and hypertension [9,10], plays a significant role in maternal IAV pathogenesis.

The PVAT is a component of the aorta, part of the tunica externa, which also houses the periadventitial space and these are paramount for vascular function. The aorta is also comprised of two additional structural layers, which are regulators of its function: Tunica intima, the inner most layer housing the endothelium, and the tunica media—the smooth muscle [11]. The endothelium is a single cell layer that physiologically functions to regulate vessel tone, vascular homeostasis, neutrophil recruitment, and platelet and leukocyte interactions [12]. Moreover, the endothelial cells release vasoactive factors that function locally to dilate or constrict smooth muscle cells within arterial walls, to regulate blood flow and in small resistance blood vessels, the blood pressure [13,14]. The endothelium and smooth muscle, are well regarded to influence vascular pathogenesis, due to their roles in channelling and signalling vasoactive NO [15]. Nevertheless, in vascular pathologies such as hypertension, the presence of immune cells, adhesion molecules and oxidative stress, creates an inflammatory milieu in the vasculature that dampens NO availability leading to vascular/endothelial dysfunction [16]. Human and vascular disease preclinical animal models have focused mainly on endothelial inflammation as a key initiator of vascular endothelial dysfunction, but only more recently, has the contribution of the PVAT in the pathogenesis been considered.

Similar to the endothelium, the PVAT directly regulates vascular tone via the release of key substances including adipocyte derived relaxing factors (ADRFs) that modulate vessel function and homeostasis [17]. Anatomically, PVAT surrounds blood vessels and shares some morphological and functional similarities with brown adipose tissue (BAT) such as the regulation of metabolic activities [10,18]. The prototypical role of the PVAT was mechanical support of the vessel [17,19], but recent evidence extends the role of the PVAT into an immune mechanistic role. For example, the PVAT contributes to the modulation of vascular function in chronic disease states by contributing to inflammation and immune activation [10]. In atherosclerotic apolipoprotein E^-/-^ (APOE^-/-^) mice, PVAT inflammation precedes atherosclerotic plaque formation and the development of oxidative stress and endothelial dysfunction [7]. Moreover, in hypertension, the PVAT initiates endothelial inflammation by accumulating and activating immune T cells and triggering oxidative stress to drive endothelial dysfunction [20–22]. A key mechanism of how the PVAT drives chronic vascular disease is via the release of paracrine factors [10] including the pro-inflammatory cytokines, tumour necrosis factor α (TNF-α) and interleukin 6 (IL-6), both of which negatively affect the vascular smooth muscle cells (VSMCs) and endothelial cells, resulting in the initiation of vascular inflammation [16,18]. This inflammatory PVAT phenotype serves as an important trigger for endothelial dysfunction. Although the vascular inflammatory characteristics occurring in chronic disease states such as hypertension are reminiscent of those observed in our IAV-infected pregnancy mouse model [6], the pathological involvement of the PVAT in IAV-induced endothelial dysfunction in pregnancy remains unknown.

In the present study, we utilised a well-characterised pregnant mouse model of IAV infection to establish: (i) whether IAV infects the PVAT of the aorta following intranasal infection in mice; (ii) if the immune cell profile within the vessel and PVAT and immune T cell activation is occurring within the PVAT in a manner analogous to that observed in hypertension. We show IAV disseminates into the PVAT at 1 day post infection (d.p.i) and this was at a significantly greater magnitude than that observed in the arterial wall. Endothelial dysfunction of the aorta was observed as early as 1 d.p.i. Pro-inflammatory monocytes and neutrophils infiltrated the PVAT of the aorta and to a lesser degree the wall of the aorta, however, the monocyte response occurred firstly within the vessel wall. Furthermore, a predominant infiltration and activation of T cells in the PVAT occurred following IAV infection without any discernible T cell homing to the arterial wall. This suggests that during pregnancy the PVAT is a critical vascular niche for IAV infection, and culminates in an exacerbated inflammatory vascular immune response that can cause profound downstream pathogenesis.

## Results

### Influenza A virus infection in pregnancy results in greater viral dissemination to perivascular adipose tissue versus vessel wall

**Fig 1:**
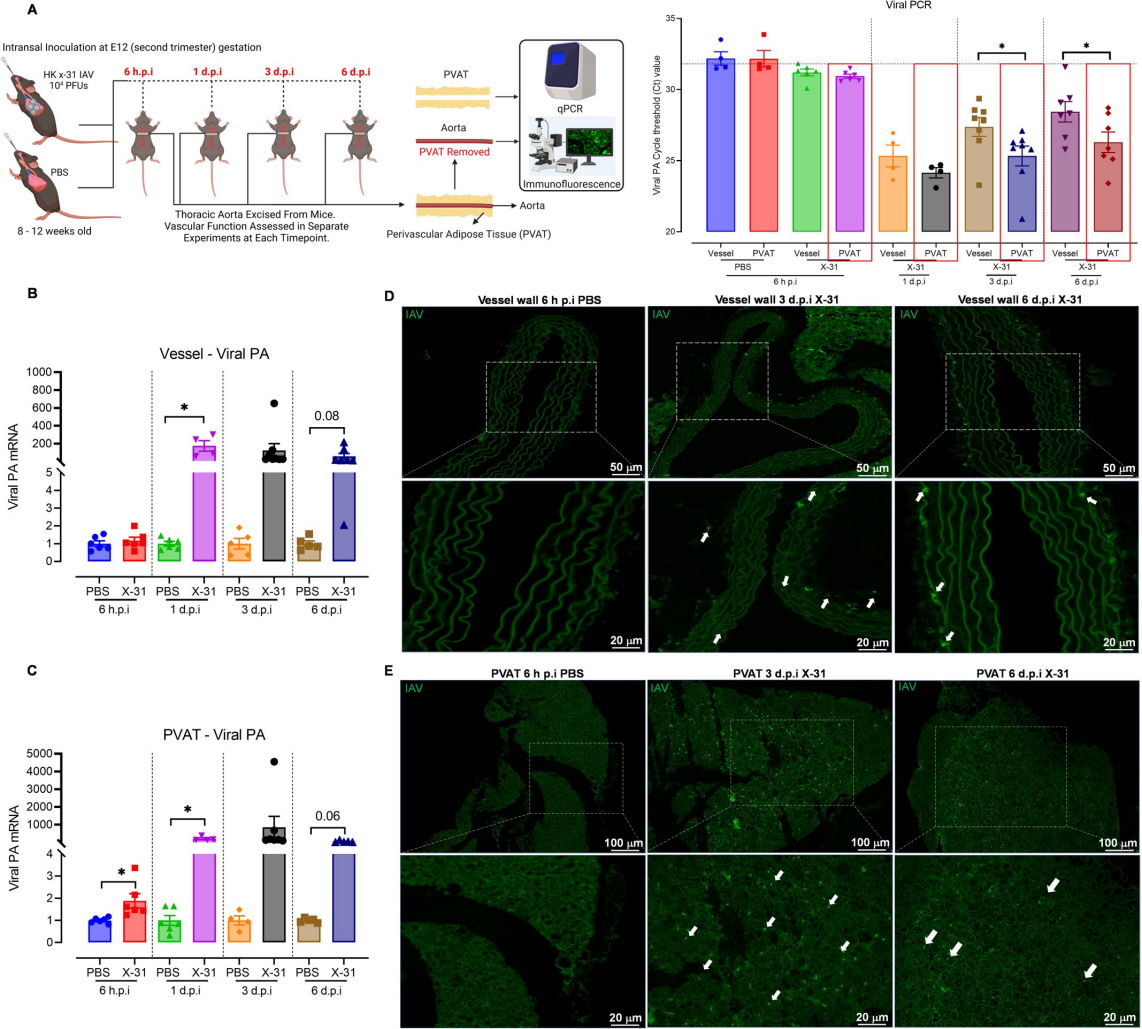
Influenza A virus (IAV) primarily disseminates into the PVAT of pregnant mice compared to the arterial wall. Eight-to-twelve-weeks-old pregnant (E12 gestation) C57BL/6 mice were intranasally inoculated with PBS or Hk-x31 (X-31; 10^4^ PFU) for arterial wall and PVAT tissue assessment at 6 hours post infection (h.p.i), 1, 3 and 6 days post infection (d.p.i). (A) Schematic of infection schedule and experiments (created with BioRender.com), the presence of IAV burden in the arterial wall and PVAT was confirmed through qPCR, using a cycle threshold of <31 cycles as a confirmed infection. (B—C) Vessel wall and PVAT gene expression of viral PA normalized to GAPDH or RPS18. (D) Representative immunofluorescence image of the arterial wall of pregnant PBS and Hk-x31 infected mice at 6 h, or 3 and 6 d.p.i labeled with IAV nucleoprotein antibody (green). (E) Representative immunofluorescence image of the PVAT of pregnant PBS at 6 h.p.i and Hk-x31 infected mice at 6 h, or 3 and 6 d.p.i labeled with IAV nucleoprotein antibody (green). Data are represented as mean ± SEM (pregnant PBS, n = 4–8; pregnant X-31, n = 4–8 of at least two to three independent experiments). All fold change calculations of the X-31 group were measured via qPCR, performed against the PBS group within its respective timepoint and normalised against RPS18 (except otherwise stated). Statistical analysis was performed using unpaired t-test against their respective PBS control. * *P*<0.05.

We recently reported that IAV disseminates into the aorta to trigger a vascular inflammatory response and endothelial dysfunction in pregnant mice [6]. The detection of IAV mRNA transcripts in maternal thoracic aorta at 3 d.p.i prompted us to investigate the distribution of IAV into the arterial wall and PVAT of the aorta. Viral dissemination was measured by real-time PCR to detect the presence of viral polymerase acidic protein (PA) mRNA. Samples that had Ct values less than 31 was considered IAV positive. In the arterial wall, detection of viral PA indicated that IAV dissemination occurred at 1 d.p.i (Fig 1A). Viral PA mRNA load was consistently significantly greater in the PVAT than compared to the arterial wall at 1, 3 and 6 d.p.i (Fig 1A and S1 Table). The Ct values between PVAT and arterial wall accounts for ~4–8 fold higher expression within the PVAT. Viral PA Ct values were further analysed by normalising to a housekeeping gene and assessed for statistical differences. Notable statistical differences and trends were observed in both the arterial wall and PVAT (Fig 1B and 1C). IAV dissemination was confirmed using immunofluorescence for detection of IAV nucleoprotein (NP). NP was detected within the arterial wall as well as the periadventitial space at 1, 3 and 6 d.p.i (Figs 1D and S1). Interestingly, in the PVAT, IAV dissemination also occurred as early as 1 d.p.i (S2 Fig) but no discernible elevation at 6 h post-infection (h.p.i). IAV NP was also detected in the PVAT at 3 and 6 d.p.i, with peak detection at 3 d.p.i and a subsequent decline in viral NP at 6 d.p.i (Fig 1E). Interestingly, qPCR analysis of viral load in the heart tissue revealed a significant increase in IAV dissemination to the heart. This finding suggests that the heart could be significantly impacted by IAV infection in pregnancy, but this warrants further investigation (S4 Fig). Therefore, IAV accumulates to a greater degree in the PVAT versus the arterial wall, suggesting that the PVAT offers a more conducive environment for the virus than the arterial wall.

### Influenza A virus infection during pregnancy drives early onset endothelial dysfunction

**Fig 2:**
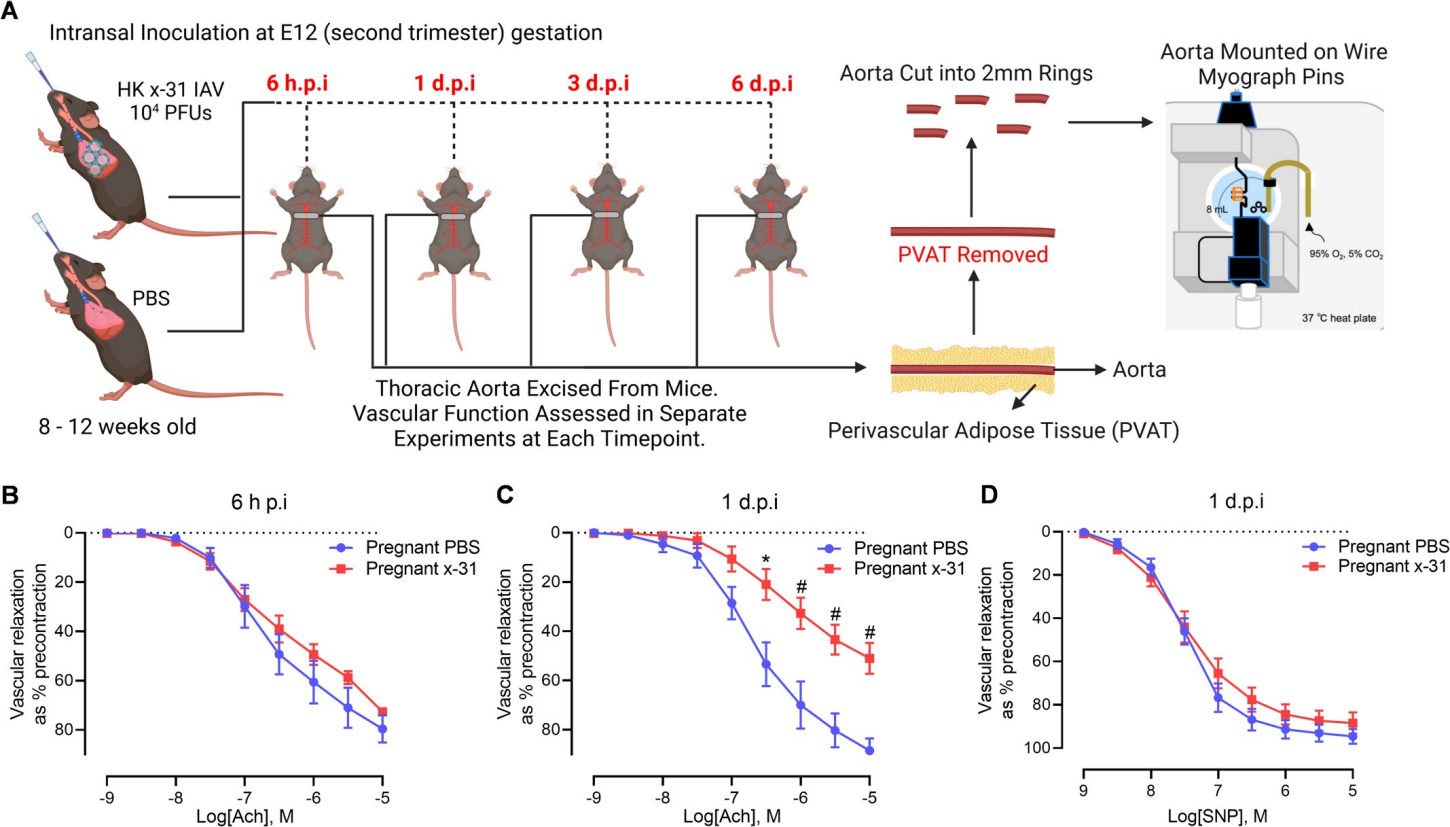
IAV infection triggers early onset endothelial dysfunction in pregnant mice. Vascular function was assessed at 6 h and 1 d.p.i in isolated thoracic aortic rings of pregnant mice inoculated with PBS or Hk-x31 (X-31; 10^4^ PFU). (A) Schematic of infection schedule and experiments (created with BioRender.com) (B) Endothelium-dependent vasodilation to acetylcholine (ACh) at 6 h.p.i. (C) 1 d.p.i vascular function assessment to endothelium-dependent vasodilator—ACh. (D) 1 d.p.i vascular reactivity assessment to endothelium independent vasodilator–SNP. Vascular relaxation is calculated as a % of pre-constriction to U-46619 (thromboxane agonist). Data are represented as mean ± SEM (pregnant PBS, n = 4–8; pregnant X-31, n = 4–8 of at least two independent experiments). Statistical analysis was conducted using a two-way analysis of variance (ANOVA) followed by Holm’s Sidak post-hoc multiple comparison. * P<0.01, #*P*<0.001.

Given that IAV disseminates as early as 1 d.p.i into the arterial wall and PVAT, we next performed functional assessments of the aorta using wire myography to establish if the presence of viral mRNA is associated with vascular dysfunction. Vascular endothelial and smooth muscle function was accessed by either the endothelium-dependent vasodilator acetylcholine (ACh), or the endothelium-independent vasodilator, sodium nitroprusside (SNP). At 6 h.p.i, vascular functional responses to ACh remained similar to the responses of uninfected controls (Fig 2B). However, at 1 d.p.i, endothelium-dependent vasorelaxation to ACh (Fig 2C) was significantly impaired while there was no alteration in the endothelium-independent vasodilation to SNP (Fig 2D). This finding suggests that the dissemination of IAV into the PVAT and arterial wall as early as 1 d.p.i, is associated with an early onset of endothelial dysfunction.

### Influenza A virus triggers inflammation and oxidative stress in the arterial wall and perivascular adipose tissue

**Fig 3:**
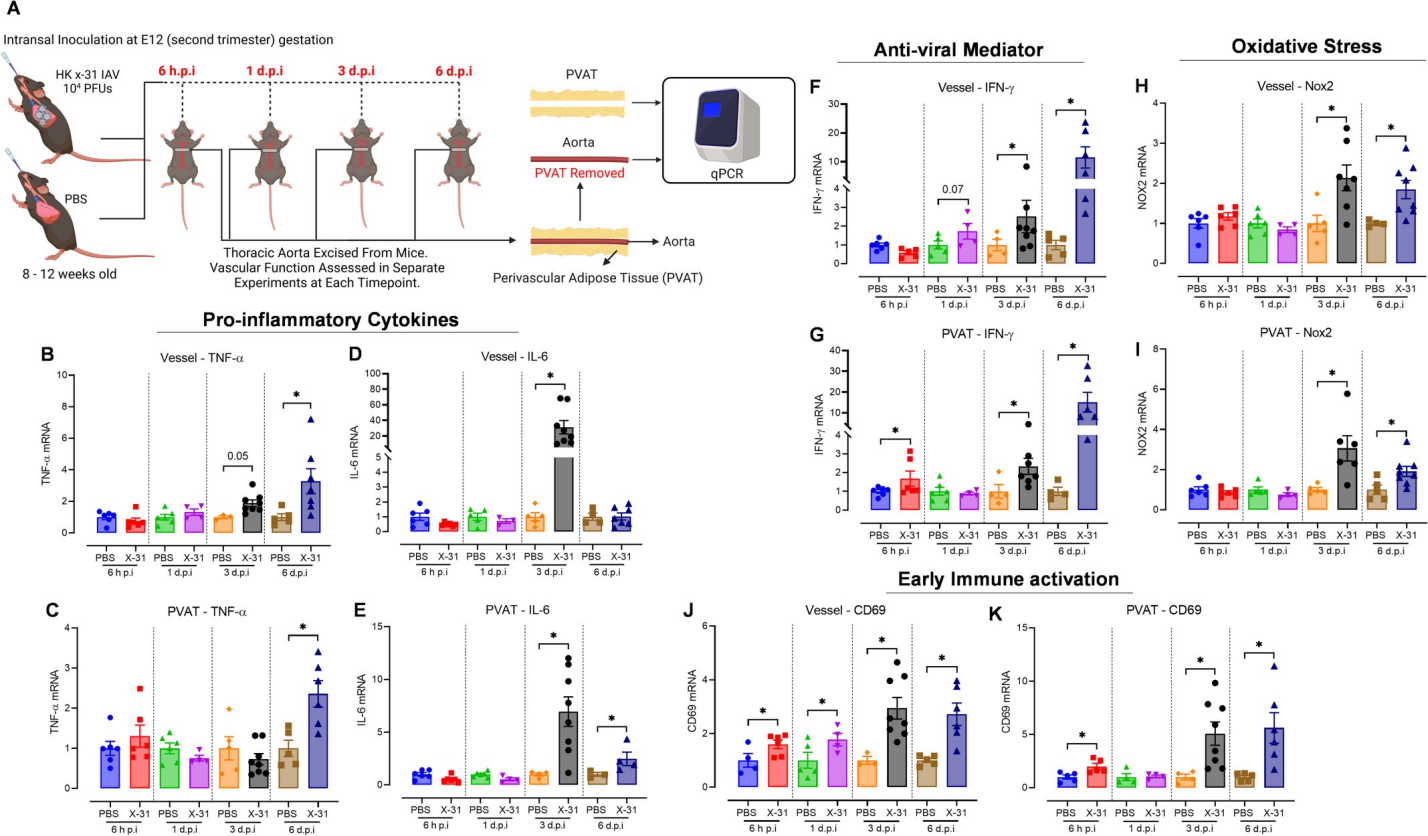
IAV induces an inflammatory and oxidative stress phenotype in both the arterial wall and PVAT in pregnant mice. Eight-to-twelve-week-old pregnant (E12 gestation) C57BL/6 mice were intranasally inoculated with PBS or Hk-x31 (X-31; 10^4^ PFU) for vessel wall and PVAT tissue inflammatory and oxidative stress mRNA transcripts assessment at 6 h, or 1, 3 and 6 d.p.i. (A) Schematic of infection schedule and experiments (created with BioRender.com) (B—E) Vessel wall and PVAT gene expression of pro-inflammatory cytokines TNF-α and IL-6. (F—G) Antiviral mediator IFN-γ mRNA transcript gene expressions as determined via qPCR in the vessel wall and PVAT. (H—I) Vessel wall and PVAT gene expression of oxidative stress marker NOX2. (J—K) Early immune activation marker CD69 mRNA transcript gene expressions as determined via qPCR in the vessel wall and PVAT. Data are represented as mean ± SEM (pregnant PBS, n = 4–8; pregnant X-31, n = 4–8 of at least two independent experiments). All fold change calculations of the X-31 group were measured via qPCR, performed against the PBS group within its respective timepoint and normalised against RPS18. Statistical analysis was performed using unpaired t-test against their respective PBS control. * *P*<0.05.

Pro-inflammatory cytokine production and oxidative stress are important contributors to vascular pathologies, which underpin systemic vascular alterations in pregnant mice [6]. Therefore, we assessed whether IAV triggers an inflammatory response in the arterial wall and PVAT prior to endothelial dysfunction. In the vessel wall, we observed no significant differences in pro-inflammatory cytokines TNF-α, IL-6, IFN-γ as well as oxidative stress marker, NADPH oxidase 2 (NOX2) at the 6 h.p.i and 1 d.p.i time points (Fig 3B–3E and 3H–3I). However, the early pan leukocyte activation marker—CD69 was significantly elevated as early as 6 h.p.i with persistent elevation observed at 1, 3 and 6 d.p.i in the arterial wall (Fig 3J).

When we examined the arterial wall phenotype at 3 d.p.i. we noted that there was a trend towards a significant increase (*P = 0*.*05*) in the pro-inflammatory cytokine TNF-α, which was then significantly increased at 6 d.p.i (Fig 3B). IL-6 was only significantly elevated at 3 d.p.i (Fig 3B) with a complete reversal of its expression by 6 d.p.i (Fig 3D). The expression of IFN-γ increased at 3 and 6 d.p.i (Fig 3F). Furthermore, the NOX2 gene was significantly upregulated at 3 d.p.i and maintained at 6 d.p.i (Fig 3H). Anti-inflammatory adipokine adiponectin was unchanged in the arterial wall until 6 d.p.i (S3 Fig).

In the PVAT, at the earliest time point assessed i.e., 6 h.p.i and prior to endothelial dysfunction there was a significant but small elevation in CD69 and IFN-γ but no change in TNF-α, IL-6, or NOX2 (Fig 3). TNF-α was only significantly increased at 6 d.p.i (Fig 3C); IL-6 was significantly increased at 3 and 6 d.p.i (Fig 3E); IFN-γ and NOX2 were significantly increased at 3 and 6 d.p.i, (Fig 3G and 3I) and CD69 showed persistent elevation over the 6 days of infection (Fig 3K). These findings suggest that the initial trigger for endothelial dependent vascular dysfunction was unlikely to be a direct consequence of an overt pro-inflammatory and oxidative response, but possibly due to a virus dependent IFN-γ and CD69 response that occurred in the PVAT and in the vascular wall.

### Maternal influenza A virus infection promotes inflammatory monocytes and neutrophilic cell infiltration into the perivascular adipose tissue

**Fig 4:**
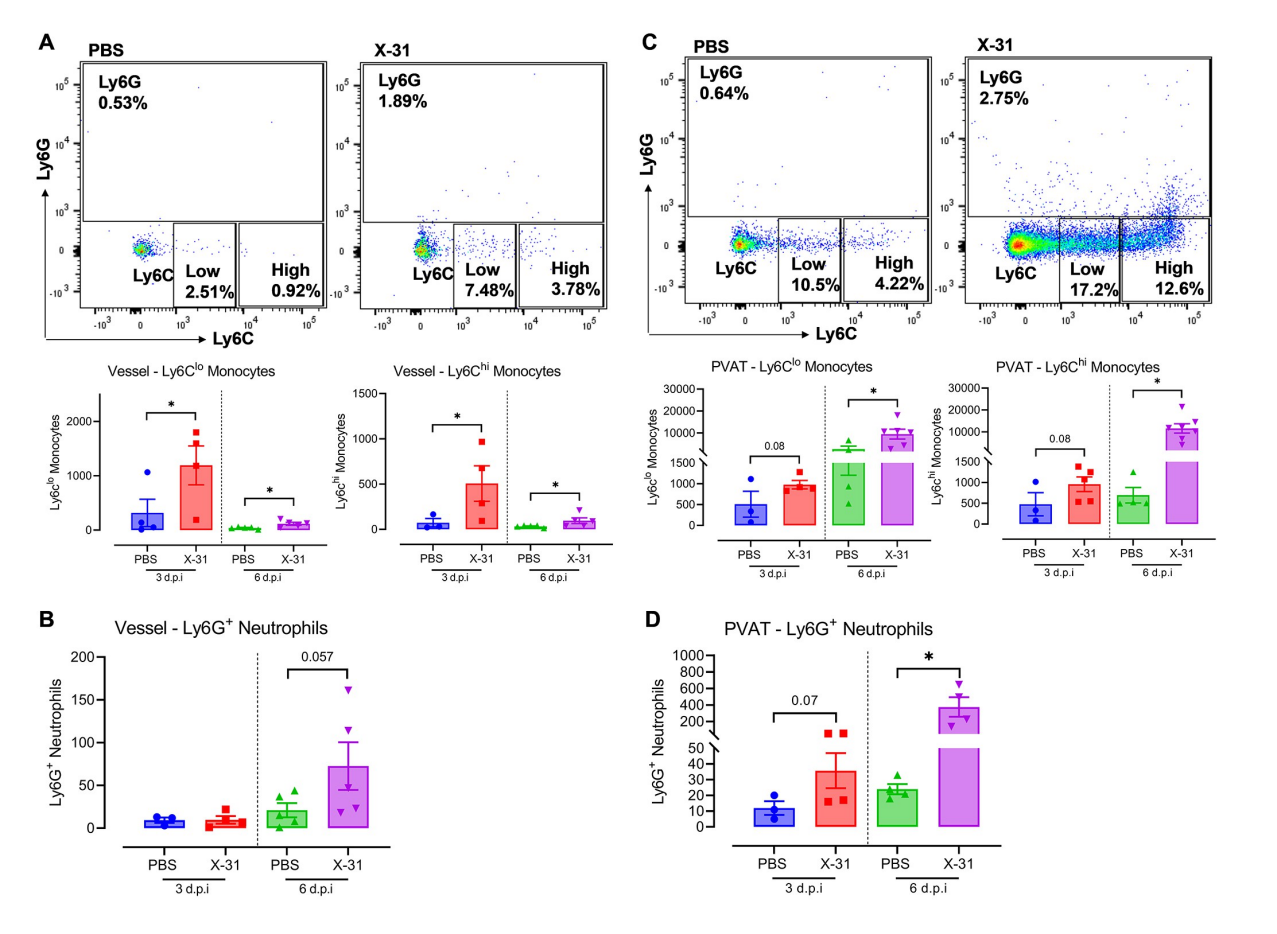
IAV promotes innate inflammation via substantial infiltration of monocytes and neutrophils in PVAT compared to arterial wall in pregnant mice. Separate single cell suspensions were prepared from vessel wall and PVAT digests from pregnant mice that were inoculated with either PBS or Hk-x31 virus (X-31; 10^4^ PFU) at 3 and 6 d.p.i and quantified via flow cytometry for the following cell subsets. (A) Representative dot plots and quantification showing patrolling monocytes (CD11b^+^Ly6C^low^) and pro-inflammatory monocytes (CD11b^+^Ly6C^high^) in the vessel wall. (B) Ly6G^+^ neutrophils quantification. Quantification results in the vessel wall are also shown. (C) Representative dot plots and quantification showing patrolling monocytes (CD11b^+^Ly6C^low^) and pro-inflammatory monocytes (CD11b^+^Ly6C^high^) in the PVAT. (D) Ly6G^+^ neutrophils quantification. Quantification results in the PVAT are also shown. All cell populations were measured as absolute number of CD45^+^ population per 25,000 counting beads. Data are represented as mean ± SEM (pregnant PBS, n = 4–6; pregnant X-31, n = 4–6; of at least two independent experiments). Statistical analysis was performed using unpaired t-test against their respective PBS control. * *P*<0.05.

We next investigated the infiltration of monocytes and neutrophils into the arterial wall and PVAT as a means to examine the innate vascular immune response. In the arterial wall, CD11b^+^Ly6C^low^ ‘patrolling’ monocytes and ‘pro-inflammatory’ CD11b^+^Ly6C^high^ monocytes were significantly elevated by IAV infection at both 3 and 6 d.p.i (Fig 4A). There was also a trend towards a significant increase (*P = 0*.*057*) in CD11b^+^Ly6G^+^ neutrophils at 6 d.p.i (Fig 4B). In contrast, in the PVAT, there was no significant effect of IAV infection on CD11b^+^Ly6C^low^ *(P = 0*.*08)* and CD11b^+^Ly6C^high^ *(P = 0*.*08)* monocytes at 3 d.p.i (Fig 4C). However, at 6 d.p.i, the number of CD11b^+^Ly6C^low^ and CD11b^+^Ly6C^high^ monocytes were significantly and substantially higher in the PVAT compared to the arterial wall (Fig 4C). Inflammatory CD11b^+^Ly6G neutrophils were also significantly elevated in the PVAT at 6 d.p.i, with an increasing but insignificant *(P = 0*.*07)* trend observed at 3 d.p.i (Fig 4D). Therefore, IAV promotes a monocyte and neutrophil based innate immune response that occurs firstly in the arterial wall but with a delayed and ultimately substantially greater response in the PVAT.

### The infiltration of activated T cell phenotypes occurs predominantly in the perivascular adipose tissue compartment

**Fig 5:**
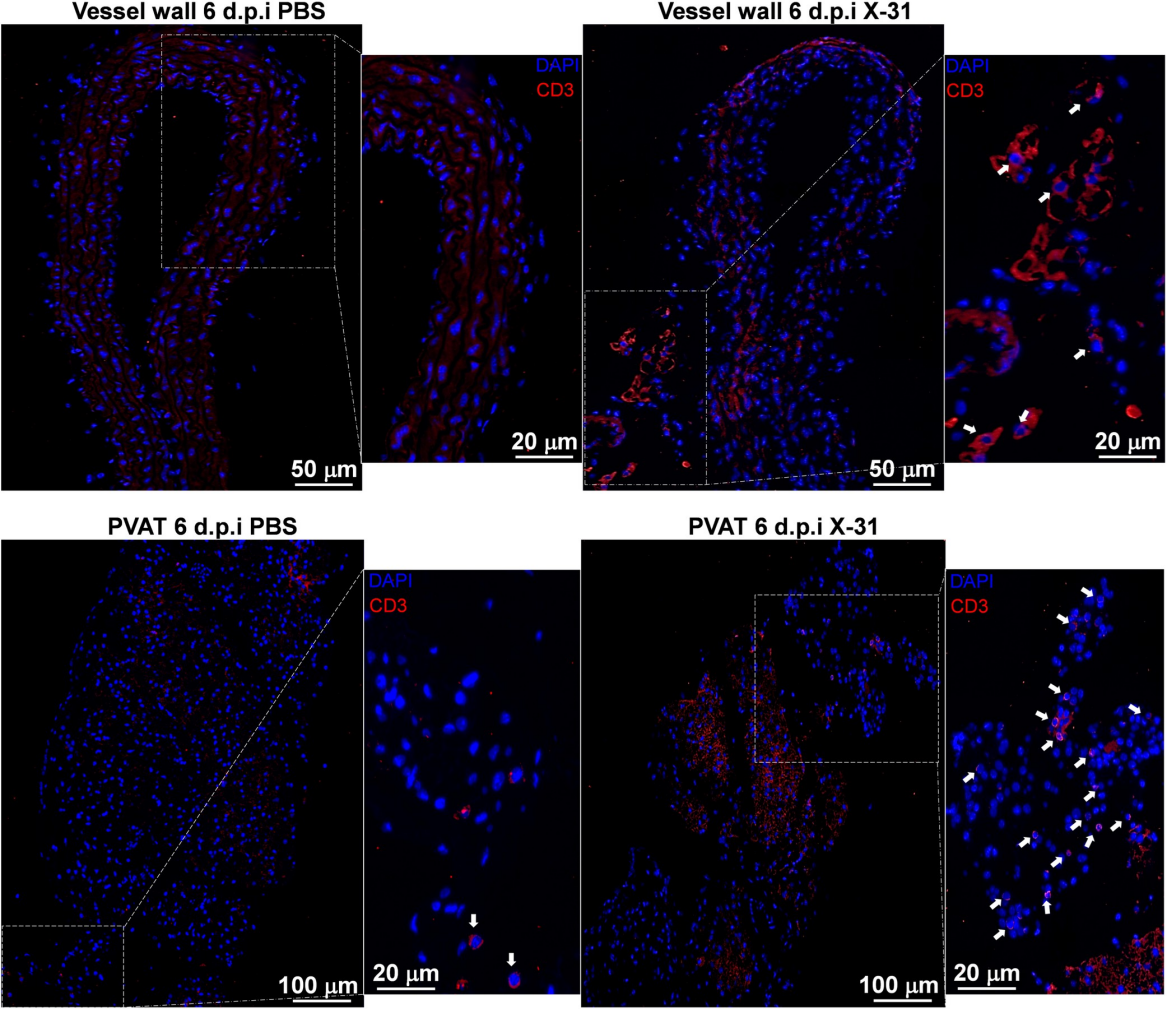
IAV drives an increase in global CD3^+^ immune T cell infiltration and activation predominantly in the PVAT and periadventitial space of pregnant mice when compared to the arterial wall. Representative Immunofluorescence image of pregnant PBS and X-31 mice arterial wall and PVAT labelled with CD3 antibody (red) and counterstained with DAPI (blue). Data are representative of pregnant PBS, n = 5–6; pregnant X-31, n = 4–6; of at least two independent experiments.

**Fig 6:**
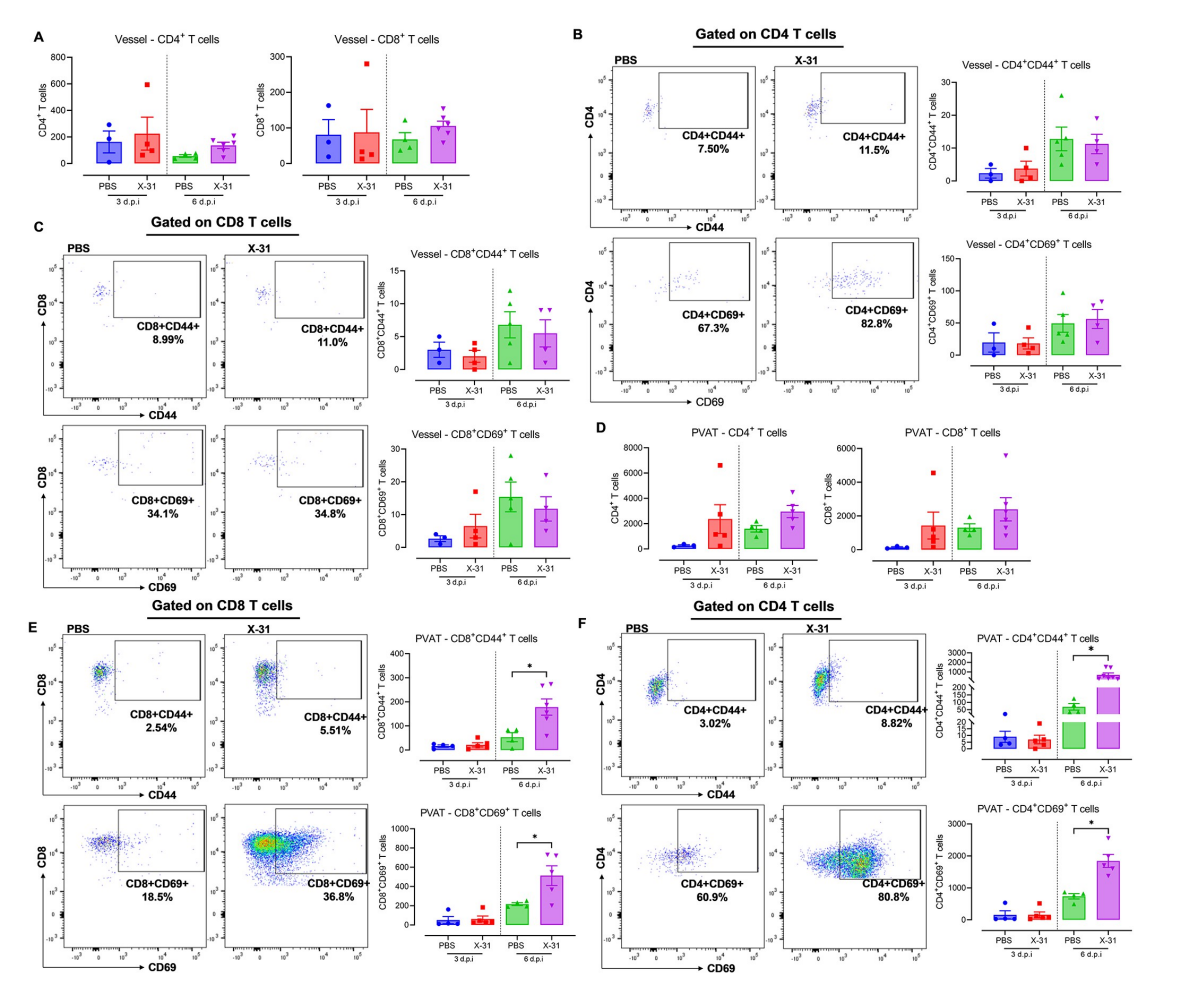
IAV drives immune T cell infiltration and activation predominantly in the PVAT of pregnant mice when compared to the arterial wall. Separate single cell suspensions were prepared from vessel wall and PVAT digests from pregnant mice that were inoculated with either PBS or Hk-x31 virus (X-31; 10^4^ PFU) at 3 and 6 d.p.i and quantified via flow cytometry for the following cell subsets. (A) Vessel wall analysis showing CD4^+^ and CD8^+^ T cells at 3 and 6 d.p.i. (B—C) Representative X-31 dot plots and quantification showing PBS and X-31 CD44^+^ and CD69^+^ activated CD4^+^ and CD8^+^ T cells in the vessel wall. (D) PVAT analysis showing CD4^+^ and CD8^+^ T cells at 3 and 6 d.p.i. (E—F) Representative X-31 dot plots and quantification showing PBS and X-31 CD44^+^ and CD69^+^ activated CD4^+^ and CD8^+^ T cells in the PVAT. All cell populations are measured as absolute number of CD45^+^ population per 25,000 counting beads. Data are represented as mean ± SEM (pregnant PBS, n = 3–6; pregnant X-31, n = 4–6; of at least two independent experiments). Statistical analysis was performed using unpaired t-test against their respective PBS control. * *P*<0.05.

Given that T cells influence vascular function during hypertension and atherosclerosis, and as more recently shown, in IAV-induced vascular dysfunction in pregnant mice [6,8,21], the extent to which aortic compartment (PVAT or the arterial wall) drives the majority of T cell responses in response to IAV infection was investigated. We assessed CD3^+^ T cell populations *via* immunofluorescence staining and observed a significant increase in CD3^+^ T cells in the PVAT and the surrounding periadventitial space (Fig 5). There was no detectable CD3^+^ staining in the arterial wall. Using flow cytometry, in the arterial wall, there was no alteration in CD4^+^ and CD8^+^ T cell (Fig 6A). A similar trend was observed with their CD44^+^ and CD69^+^ activated forms at 3 and 6 d.p.i (Fig 6B and 6C). In contrast, in the PVAT, although there were no significant increases in CD4^+^ and CD8^+^ T cells (Fig 6D), their activated forms were significantly altered. For instance, whilst the CD4^+^CD44^+^ and activated CD4^+^CD69^+^ T cells remained unaltered by IAV infection at 3 d.p.i, at 6 d.p.i the number of CD4^+^CD44^+^ and activated CD4^+^CD69^+^ T cells were significantly higher (Fig 6E). Similarly, CD8^+^CD44^+^ and activated CD8^+^CD69^+^ T cells infiltrated the PVAT with significant elevations observed at 6 d.p.i, but with no alterations at 3 d.p.i, which corroborates with our previous study [6] where the adaptive immune response was prevalent at 6 d.p.i in maternal aorta (Fig 6F). This suggests that IAV drives a significant immune T cell infiltration and activation predominantly in the PVAT.

## Discussion

This study is the first to characterise the inflammatory and immune cell profile of the arterial wall and PVAT in pregnant mice following IAV infection. We show that the PVAT is susceptible to IAV infection with substantially higher levels of IAV mRNA and viral antigens compared to the arterial wall. Moreover, IAV infection induced the expression of the anti-viral mediator IFN-γ, which was associated with vascular endothelial dysfunction at 1 d.p.i. We also identified an influx of pro-inflammatory monocytes and neutrophils to the PVAT following infection, and a preferential infiltration of CD4^+^ and CD8^+^ T cells to the PVAT. Collectively, these data suggest that the PVAT is an essential site for viral inflammation during pregnancy and the subsequent monocyte and T cell accumulation that ensues in response to IAV infection. The evidence highlights a pro-inflammatory role of the PVAT in the initiation and development of IAV induced vascular pathology, which shares similar pathological inflammatory features with other vascular diseases such as hypertension and atherosclerosis.

Direct IAV infection of the aorta is not exclusive to pregnancy, but the vascular pathology is markedly exacerbated during pregnancy. Different IAV viral strains have been shown to disseminate and infect the aorta independent of sex [23]. Despite this, vascular inflammation occurs at a much lower rate in non-pregnant compared to pregnant mice [6,23]. The vascular pathogenesis that befalls pregnant and non-pregnant mice following IAV infection of the aorta is vastly different. This is due to the more profound inflammatory response and viral burden that occurs in pregnant mice [6]. Moreover, endothelial dysfunction in response to IAV infection appears to occur almost exclusively in pregnant mice. The substantial localisation of IAV mRNA transcripts and viral antigens to the PVAT in comparison to the arterial wall, signifies that there is preferential trafficking of IAV to this vascular compartment. IAV localisation to the PVAT in the aorta is likely to be detrimental to aortic function, due to the critical role of the PVAT in maintaining normal vascular haemostasis in a non-pathogenic state [24]. For instance, physiologically, the PVAT regulates vascular tone and the regulation of blood vessel function through the release of vasoactive factors such as NO and ADRF [17]. A dysfunctional PVAT can negatively impact on blood vessel function leading to enhanced contractile responses that are characteristic in hypertension [25]. Indeed, in disease models of atherosclerosis and hypertension, the PVAT undergoes substantial cellular and molecular alterations that initiates a switch from a protective to a pathogenic role [10]. In the present study the dissemination of greater levels of IAV to the PVAT suggests that this event could potentially prime the aorta to become dysfunctional, as the infection progresses. Indeed, at 1 d.p.i, there was a significant impairment of the endothelial-dependent vasorelaxation response to ACh in the aorta. This suggests that IAV dissemination into the PVAT results in a reduction in bioavailability of endothelial derived NO culminating in a supressed vascular relaxation response. However, despite the predominance of virus in the PVAT, the overall inflammatory immune response to IAV infection is likely to be the result of a dynamic balance of effects at the PVAT and the arterial wall, with different elements of the immune response being variably activated in one or the other compartment. Ultimately the viral load appeared higher in the PVAT and while some of the cellular immune response (e.g. CD69, monocyte, neutrophil and oxidative stress) was higher in the PVAT the reverse was true for the pro-inflammatory IL-6 and TNF-α responses with higher expression in the vessel wall.

Historically, the effects of IAV on vascular function and on the physiological cardiovascular adaptations that occur during pregnancy have not been thoroughly examined. Nonetheless, our recent study highlighted for the first time the critical effects of IAV on large blood vessel function [26]. Our study demonstrated that IAV infects the aorta in pregnant mice to trigger an inflammatory cascade, which modifies the maternal vascular landscape at 3 and 6 d.p.i [6]. However, how early aortic IAV dissemination and vascular dysfunction occurred was not investigated. Here, the observed impairment in endothelium-dependent relaxation at 1 d.p.i might have occurred as a result of direct IAV infection, causing apoptosis of the endothelial cells, and/or via the increase in IFN-γ [27]. Increased IFN-γ stimulation in blood vessels could lead to endothelial cell apoptosis resulting in vascular dysfunction [27–29]. The recruitment of IFN-γ is suggested to precede the development of atherosclerosis [30]. Although IFN-γ has also been associated with the modulation of VSMC constriction, the effect of IFN-γ on smooth muscle relaxation was only observed at the 3 d.p.i (~40%) timepoint in the seminal study [6]. This is comparable to the vascular pathology observed in atherosclerosis where endothelial dysfunction occurs first, and VSMC impairment typically occurs at an advanced phase in atherosclerosis development [29].

An established and widely accepted concept is that chronic low-grade inflammation exhibits some of the pathological hallmarks of cardiovascular diseases, such as atherosclerosis and hypertension, which are triggered by an array of altered phenotypic and structural factors in the blood vessel [31,32]. Of significance, PVAT dysfunction which is characterised by increased cytokines, chemokines and oxidative stress burden, has been shown to drive chronic vascular inflammation [10]. The inflammatory phenotype that develops in a dysfunctional PVAT, significantly alters its key function in vascular tone regulation [17]. This concept is extensively studied in atherosclerotic and hypertensive disease, where the imbalance in vasoactive factors and the recruitment of pro-inflammatory immune cells results in endothelial dysfunction [7,20–22]. In an IAV infected aorta, the vascular inflammation that occurs, entails contributions from the PVAT and the arterial wall. In the PVAT, despite the increase in IFN-γ at 6 h.p.i, the pathological consequence on the aorta remained undetectable until 1 d.p.i. Along with pro-inflammatory cytokines, oxidative stress is concomitantly triggered by IAV infection and is a key influence in vascular pathology [33]. Oxidative stress, which is characterised by the over production of reactive oxygen species (ROS) and superoxide anions, promotes endothelial dysfunction in vascular diseases [34,35]. IAV infection has been shown to induce oxidative stress in the lungs [34] and more recently in the aorta of pregnant mice [6]. NOX2 is the catalytic subunit of the prototypical NADPH oxidase that specifically localises to endosomes/phagosomes and is a major source of ROS during IAV infection ^35^. NOX2 expression was significantly elevated in the arterial wall and PVAT at 3 and 6 d.p.i in corroboration with our previous study [6]. Despite this transcriptional increase in NOX2, further investigations are warranted to discern the impact of ROS on vascular function during the early stages of IAV infection in pregnant mice.

The impact of monocytes on the pathogenesis of inflammatory and cardiovascular disease conditions cannot be overstated [36,37]. Insights into the inflammatory burden of IAV infection and the link to an altered cardiovascular landscape has recently become a major area of interest. The “vascular storm”, which destabilises maternal vasculature during IAV infection is associated with extensive infiltration and activation of monocytes and neutrophils. The increase in the recruitment and infiltration of patrolling Ly6C^low^ monocytes and inflammatory Ly6G^+^ neutrophils during IAV infection in this study is suggested to contribute to focal necrosis of infected endothelial cells [6,38]. Moreover, it is well documented that the accumulation of Ly6C^low^ monocytes and Ly6G^+^ neutrophils at the site of infection assists in facilitating the clearance of pathogens [38]. In this study, the accumulation of Ly6C^low^ monocytes and Ly6G^+^ neutrophils to the PVAT following IAV infection signifies an important innate immune response in an attempt to initiate IAV clearance. Similarly, the increased pro-inflammatory Ly6C^high^ monocytes in both the PVAT and arterial wall is evidence of an enhanced viral clearance at the infection site [39]. This process ensues irrespective of the PVAT showing a higher inflammatory burden when compared to the arterial wall. This influx of innate immune cells to different compartments of the aorta is reflected by the preferential dissemination of IAV to the PVAT compared to the arterial wall. Irrespective of the larger IAV burden in the PVAT, the inflammatory profile in both compartments suggests that monocyte recruitment and the trafficking of innate immune cells initially occurs in the arterial wall possibly via the bloodstream prior to entry into the PVAT. Secondary innate immune cell trafficking into the PVAT may have occurred *via* its extensive vaso-vasorum network of capillaries. Pro-inflammatory adipokines including leptin and resistin are produced by adipocytes and can inhibit the production of anti-inflammatory adipokine adiponectin, which may limit the production of pro-inflammatory cytokines such as TNF-α and IL-6 [40,41]. In this study, adiponectin levels remained unaltered until 6 d.p.i, which could signal the development of a late anti-inflammatory phenotype. Nevertheless, this may not be sustainable, as most pro-inflammatory cytokines were still highly expressed at 6 d.p.i. The trafficking of immune cells first to the arterial wall and then the PVAT might occur *via* leptin-induced leukocyte chemotaxis and the release of adhesion molecules by resistin to facilitate adhesion of immune cells to the PVAT [41,42].

T cell infiltration of the arterial wall in hypertensive mouse models predominantly occurs from the PVAT [16], where T cells are observed to densely accumulate prior to activating a non-beneficial crosstalk with the arterial wall. This phenomenon is termed “outside-in” meaning that T cells infiltrate and activate within the PVAT prior to migrating into the arterial wall [16]. During IAV infection, the outside-in phenomenon may also occur due to IAV preferentially disseminating into the PVAT. Immunofluorescence staining revealed CD3^+^ T cell populations were largely present in the PVAT and periadventitial space while undetected in the arterial wall in response to IAV infection. Furthermore, the PVAT had a significantly greater population of CD4^+^ and CD8^+^ T cells when compared to the arterial wall. CD4^+^ and CD8^+^ T cells expressing adhesion protein CD44^+^ and early activation marker CD69^+^ were observed to densely populate the PVAT [43]. The significant increase in CD69 gene expression at every timepoint in the arterial wall could signify the early activation of resident T cells; although this may not translate to a significant increase in CD69 protein expression. The gene expression levels in the PVAT at 6 d.p.i does however translate to increased protein expression as detailed in our previous study [6]. This is important, as the phenotype occurring during IAV infection of the PVAT mimics the T cell responses in hypertension. It appears that the preferential accumulation and infiltration of the PVAT by T cells may be in response to the larger IAV burden. Furthermore, CD3^+^ T cell detection in the periadventitial space could suggest a delay in migration to the arterial wall. Delineating whether T cells ultimately infiltrate the arterial wall rather than brewing up an inflammatory phenotype that transcends beyond the periadventitial space requires further investigation. Collectively, the preferential accumulation and infiltration of the PVAT by T cells may suggest that the PVAT is a site prone to immune T cell inflammation that may extend to the arterial wall and cause further vascular damage.

While the results in this study were mainly observational and hypothesis generating, we do show that virus disseminates to extra pulmonary sites such as the vessel, PVAT and heart in pregnancy, but the mode of dissemination is yet to be determined. In our previous study, we demonstrated that viral dissemination was not a phenomenon that’s unique to pregnancy but rather the development of vascular dysfunction, and the heightened level of viral and inflammatory burden that occurs [6]. The process involved in facilitating viral dissemination during pregnancy remains elusive and evaluating several experimental avenues to comprehend the process involved is needed. A potential mechanism may involve IAV induced damage to alveolar barrier through the disruption of epithelial cell tight junctions which can result in viral antigen escape [44]. Another mechanism may perhaps be via the blood (viremia), the lymphatic system [45], or via phagocytoses by platelets and immune cells. A limitation of this study is discerning whether replication-competent IAV directly infects immune cells which may subsequently traffic to the vessel and PVAT.

The hyperinflammatory phenotype induce by IAV infection within the maternal vascular framework is suggestive of a mechanism that enhances systemic antiviral responses during pregnancy to protect against fetal infection at the cost of increased maternal pathology. Indeed, PBMCs isolated from pregnant women have shown increased number of activated natural killer (NK) and T cells in response to IAV infection compared with non-pregnant women [46]. This study corroborates our findings of enhanced systemic immune response against circulating viruses during pregnancy and could be a potential mechanism that underlies increased IAV-associated morbidity and mortality in pregnant women [46]. This unique process along with perhaps an increase in Tregs may also contribute to the lack of vertical transmission of IAV to the fetus [6]. A mechanistic interrogation that could enhance the conclusions of this study is the lack of data examining the expression levels of antiviral restriction factor such as the interferon inducible transmembrane protein (IFITM) 3 [47]. This protein is effective in inhibiting IAV replication when overexpressed through the inhibition of the cytosolic entry of IAV into the cell cytoplasm [47]. Examining IFITM 3 expression in the vessel and the PVAT would be vital in discerning whether active replication is occurring in either compartment due to an inhibition of IFITM 3 secretion. The detection of IAV mRNA in the PVAT at 1 d.p.i suggests that IAV dissemination to the PVAT may contribute to the vascular pathology reported at 1 d.p.i. Nevertheless, it is a plausible that a direct infection of the endothelial cells within the vessel wall perhaps results in an increase in the number of activated Ly6C^low^ monocytes. An increase in activated Ly6C^low^ monocytes correlates with increased focal necrosis of endothelial cells [38] and the consequent endothelial dysfunction [6].

A potential limitation of this study is that the IAV infection was only assessed at E12 gestation (late second/early third trimester in humans), whereas a different pathological outcome might arise during first or third trimester infection. In the present study, the gestational timepoint E12 was selected as it represents an exponential phase of fetal development [48], whereby IAV infection results in the greatest risk of pregnancy complications in humans [49–52]. Indeed, data from seasonal and pandemic IAV infection in pregnant women suggest that the risk of IAV-induced complications are higher in the second and third trimester than in the first [53,54]. The second trimester also represents the phase where the greatest alterations in the cardiovascular system are occurring which are important in ensuring fetal oxygen and nutrient supply demands. Although infection at E12 may have been optimal in addressing the cardiovascular complications, it would be interesting to determine whether a similar maternal and fetal pathology arises if infection occurred at E7.5 (first trimester) or late third trimester at E17.5.

In conclusion, we provide evidence that IAV directly infects the PVAT in pregnant mice to initiate vascular dysfunction. Moreover, the PVAT is the main site for T cell infiltration and activation, which are key to facilitating inflammation and viral clearance. This study provides a fundamental insight into how the PVAT promotes vascular pathology in pregnancy during IAV infection; and has direct relevance for how respiratory viral infections cause complications in pregnancy.

## Materials and methods

### Ethics statement

All experiments were conducted according to approval obtained from Animal Experimentation Ethics Committee of the Royal Melbourne Institute of Technology University (RMIT) Animal Ethics Committee (Ethics number 1801) and in compliance with the guidelines of the National Health and Medical Research Council (NHMRC) of Australia on animal experimentation.

### Mice, virus, and infection

Pregnant (8-12wk) C57BL6/J mice were obtained from the Animal Resources Centre (ARC) Western Australia, Australia, and maintained in a 12 h light/12 h dark cycle with unrestricted access to food and water at the animal research facility (RMIT University, Bundoora, Australia). For infections, mice were sedated with isoflurane inhalation and infected intranasally at embryonic day (E)12 gestation with 10^4^ plaque forming units (pfu) of mouse adapted H3N2 virus (Hk-x31; X-31) or phosphate buffered saline (PBS, Sigma-Aldrich, USA) for controls and and culled for endpoint analysis at 6 h, 1, 3 and 6 d.p.i. Viral aliquots were provided in PBS at a concentration of 9.6 x 10^7^ pfu/milliliter (pfu/mL) by Prof. Patrick Reading (Department of Immunology and Microbiology, The Peter Doherty Institute for Infection and Immunity, University of Melbourne). Mice were then weighed and monitored daily.

### Airways inflammation and blood analysis

At study endpoints mice were euthanised at 6 h or 1, 3, and 6 d.p.i via intraperitoneal (i.p) injection of ketamine/xylazine (180 mg/kg/32 mg/kg) and organs harvested. To assess airway inflammation, the lower jaw to the top of the rib cage was incised to expose the salivary glands, which were separated to expose the surface of the trachea. A small incision was made roughly ¾ of the way up from the trachea where a sheathed 21-Gauge needle was inserted. The lung was flushed with 300–400 μL aliquots of PBS repeatedly, with the aspirate transferred to an Eppendorf tube. Cell viability assessment involved staining total bronchioalveolar lavage fluid (BALF) cells with 10 μL Acridine Orange solution (Thermofisher Scientific, USA) and quantified using a hemocytometer. Blood was retrieved by performing a cardiac puncture to obtain between 0.6–1 mL of blood. The blood was centrifuged at 10,000 x g for 10 mins at 4°C to retrieve plasma and stored at -80°C.

### Quantification of mRNA by QPCR

Maternal lung, thoracic aortic vessel and perivascular adipose tissue were harvested from pregnant mice at 6 h or 1, 3, and 6 d.p.i for RNA extraction using the RNeasy Mini kit (Qiagen) as per manufacturer’s instructions. RNA sample concentration and quality were measured using the Nanodrop one Spectrophotometer (Thermo Scientific). The cDNA synthesis was performed on 1–2 μg of total RNA using the High-Capacity cDNA Reverse Transcription Kit (Applied Biosystems, Foster City, CA, USA). Total RNA was added to a Master Mix mixture of reagents in the High-Capacity cDNA RT kit to make a final volume of 20 μL and transcribed at the following settings: 25°C for 10 min, 37°C for 120 min, 85°C for 5 min and kept at 4°C until collection using the Veriti Thermal Cycler (Applied Biosystems, USA). Quantitative polymerase chain reaction (qPCR) was then performed using the TaqMan Universal PCR Master Mix (Applied Biosystems) and analysed on Applied biosystem QuantStudio 7 Flex Real-Time PCR System (Thermofisher, Waltham, MA, USA). The PCR primers for TNF-α, IL-6, NOX2, IFN-γ, CD69 and Adiponectin were included in the Assay on-Demand Gene Expression Assay Mix (Applied Biosystems, Foster City, CA, USA). Viral titers were measured using oligonucleotide mouse sequence for the forward and reverse primer of the segment 3 PA of influenza virus using SYBR Green PCR Master Mix (Applied Biosystems). The quantitative values were obtained from the threshold cycle (Ct) number. Gene expression analysis was performed using the comparative Ct method. Each sample individual target gene expression level was normalised against GAPDH or RPS18 mRNA expression and expressed relative to the control.

### Wire myograph

Maternal thoracic aortic rings were harvested and dissected free of perivascular adipose tissue from 6 h.p.i and 1 d.p.i pregnant mice. Harvested vessels were placed in physiological carbogen (95% O_2_ and 5% CO_2_) bubbled Krebs solution (119 NaCl, 4.7 KCl, 1.17 MgSO_4_, 25 NaHCO_3_, 1.18 KH_2_PO_4_, 5.5 glucose, 2.5 CaCl_2_ in mmol/L). The thoracic artery was later cut into 2 mm rings and mounted onto two stainless steel pins on a four-channel wire myograph Krebs containing baths (Danish myo Technology (DMT), Hinnerup, Denmark). Vessels were normalised to a resting tension of 5 mN and allowed to equilibrate for 30 mins before exposure to 0.5 x 10^−3^ M of thromboxane A2 agonist U-46619 (Cayman, MI, USA) to determine maximum smooth muscle dependent vasocontraction. Endothelium nitric oxide dependent and smooth muscle nitric oxide dependent vasodilation were assessed using increasing concentrations (1 × 10^−9^ M—1 × 10^−5^ M) of Acetylcholine (ACh) and sodium nitroprusside (SNP) respectively, in a half maximally contracted aorta. All experiments were conducted in duplicates and compared to saline treated pregnant controls.

### Flow cytometry

Maternal thoracic vessel and PVAT harvested at 3 and 6 d.p.i, were minced using scissors and then treated with a digestion buffer (composition Collagenase type XI (Sigma-Aldrich), hyaluronidase (Sigma-Aldrich) and Collagenase Type I-S (Sigma-Aldrich) for 1 h at 37°C with intermittent shaking to make up a cell suspension. Cell suspensions were filtered through a 40 μm strainer, centrifuged in a refrigerated centrifuge at 400 x g and washed twice with FACS buffer. Total viable cells were then counted, resuspended in PBS, and incubated on ice for 30 min. Cells were then stained for 15 min at 4°C with antibodies and washed twice with FACS buffer. The antibody panel used for staining, and in their different multi-colour combinations were as follows: Alexa Fluor anti-CD45 (30-F11); APC anti-CD3 (145-2C11); PE-Cy7 anti-CD8 (53–6.7); BV605 anti-CD4 (RM4-5); FITC anti-Ly6C (HK1.4); APC-Cy7 anti-Ly6G (1A8); BV421 anti-CD11b (M1/70); BV650 anti-CD69 (H1.2F3); PerCP-CD44 (IM7); PE anti-FoxP3 (FJK-16s) and live/dead Aqua (Invitrogen). Following immunostaining, cells were resuspended in FACS buffer, fixed and analysed the following day on the BD LSRFortessa X-20 flow cytometry analyser with DIVA software (Becton Dickinson Biosciences). Data were analysed using FlowJo software (Tree Star, Inc.). The cells were analysed as a percentage of the CD45^+^ (live cells) and expressed in absolute numbers per 25,000 counting beads.

### Immunofluorescence microscopy

Maternal thoracic vessel and PVAT was fixed in 10% neutral buffered formalin, embedded in paraffin, and prepared in 5 μm sections by the Department of Histology (Monash University, Clayton, Australia). Tissue sections underwent immunofluorescence staining protocol. Conjugated primary antibodies used included Anti-Influenza A (NP) (Abnova; Cat # MAB5468) to detect IAV, while unconjugated primary antibodies included Anti-CD3 antibody (SP7) (Abcam; Cat # ab16669). A conjugated secondary antibody—Goat anti-rabbit IgG H&L (Alexa Fluor 594) (Abcam; Cat # ab150080) was used for anti-CD3 antibody. Tissues were imaged using Olympus S5 VS-ASW slide scanner and quantified by two separate blinded investigators via mean positive cell counts or fluorescence intensity using the Olympus cellSens Dimension Desktop Analyser. Appropriate controls were performed–including all primary and secondary antibody combinations to identify any non-specific cross reactivity.

### Statistical analysis

All data are expressed as the mean ± SEM. All comparisons were made within experimental groups and were performed by unpaired t-test or one-way ANOVA followed by a Mann Whitney test. Dose response curve analysis for vascular reactivity studies was performed using ANOVA for repeated measures followed by Holm’s Sidak post-hoc multiple comparison. Statistical tests were performed using GraphPad Prism (GraphPad Software Version 8.2, San Diego CA, USA). Statistical significance was considered at *P*<0.05.

## Supporting information

S1 Fig IAV primarily disseminates into the PVAT of pregnant mice compared to the arterial wall. Representative immunofluorescence image of the arterial wall of pregnant Hk-x31 infected mice at 6 h and 1 d.p.i labeled with IAV nucleoprotein antibody (green). Negative control used to show level of autofluorescence. Data are representative of pregnant PBS, n = 5–6; pregnant X-31, n = 5–6; of at least two independent experiments.
(TIF)

S2 Fig IAV primarily disseminates into the PVAT of pregnant mice compared to the arterial wall. Representative immunofluorescence image of the PVAT of pregnant Hk-x31 infected mice at 6 h and 1 d.p.i labeled with IAV nucleoprotein antibody (green). Data are representative of pregnant PBS, n = 5–6; pregnant X-31, n = 5–6; of at least two independent experiments.
(TIF)

S3 Fig IAV infection increases anti-inflammatory adipokine adiponectin at 6 d.p.i in pregnant mice. Pregnant mice were inoculated with PBS or Hk-x31 (X31; 10^4^ PFU) for aortic assessment at 6 h, 1, 3 and 6 d.p.i. (A) Schematic of infection schedule and experiments (created with BioRender.com). (B) Adiponectin gene expression in the vessel wall of pregnant mice (C) Adiponectin gene expression in the PVAT of pregnant mice. Data are represented as mean ± SEM (pregnant PBS, n = 6–8; pregnant X-31, n = 6–8; of at least two to three independent experiments). All fold change calculations of the X-31 group were measured via qPCR, performed against the PBS group within its respective timepoint and normalised against RPS18. Statistical analysis was performed using unpaired t-test against the respective PBS control. * *P*<0.05.
(TIF)

S4 Fig IAV disseminates to maternal heart tissue at 1 and 3 d.p.i. Pregnant mice were inoculated with PBS or Hk-x31 (X31; 10^4^ PFU) for aortic assessment at 1 and 3 d.p.i. (A) Schematic of infection schedule and experiments (created with BioRender.com). (B) Viral PA gene expression in the heart of pregnant mice. Data are represented as mean ± SEM (pregnant PBS, n = 4–6; pregnant X-31, n = 4–8; of at least two to three independent experiments). All fold change calculations of the X-31 group were measured via qPCR, performed against the PBS group within its respective timepoint and normalised against RPS18. Statistical analysis was performed using unpaired t-test against the respective PBS control. * *P*<0.05, ** *P*<0.01.
(TIF)

S5 Fig Total number of dead cells in Vessel and PVAT at 3 and 6 d.p.i. (A) Gating strategy for dead cells identification (B) Number of Dead cells in the vessel and PVAT per 25000 beads. Data are represented as mean ± SEM (pregnant PBS, n = 3–6; pregnant X-31, n = 4–6; of at least two independent experiments). Statistical analysis was performed using unpaired t-test against their respective PBS control. * *P*<0.05.
(TIF)

S6 Fig Gating strategy for thoracic aorta single cell suspension. T cells and macrophages were gated as CD3^+^ and CD11b^+^ and respectively from CD45^+^ Lymphocytes. Patrolling Ly6C^low^, pro-inflammatory Ly6C^high^ monocytes and inflammatory Ly6G^+^ neutrophils were identified within CD11b^+^ macrophages. CD3^+^ T cells were further divided into subsets T helper (CD4^+^) and cytotoxic (CD8^+^) T cells. CD44^+^ and CD69^+^ were gated within CD4^+^ and CD8^+^ T cell subsets.
(TIF)

S1 Table Viral polymerase cycle threshold (Ct) values of vessels and perivascular adipose tissue from control and infected dams from 6 h, 1, 3 and 6 d.p.i study timepoints. Low Ct value represents higher infection and replication.
(DOCX)
