# Supplementary figures and images for: Influenza A virus elicits peri-vascular adipose tissue inflammation and vascular dysfunction of the aorta in pregnant mice
Source: PLoS Pathog. 2022 Aug 5;18(8):e1010703. doi: 10.1371/journal.ppat.1010703 (PMC9385053; doi:10.1371/journal.ppat.1010703)

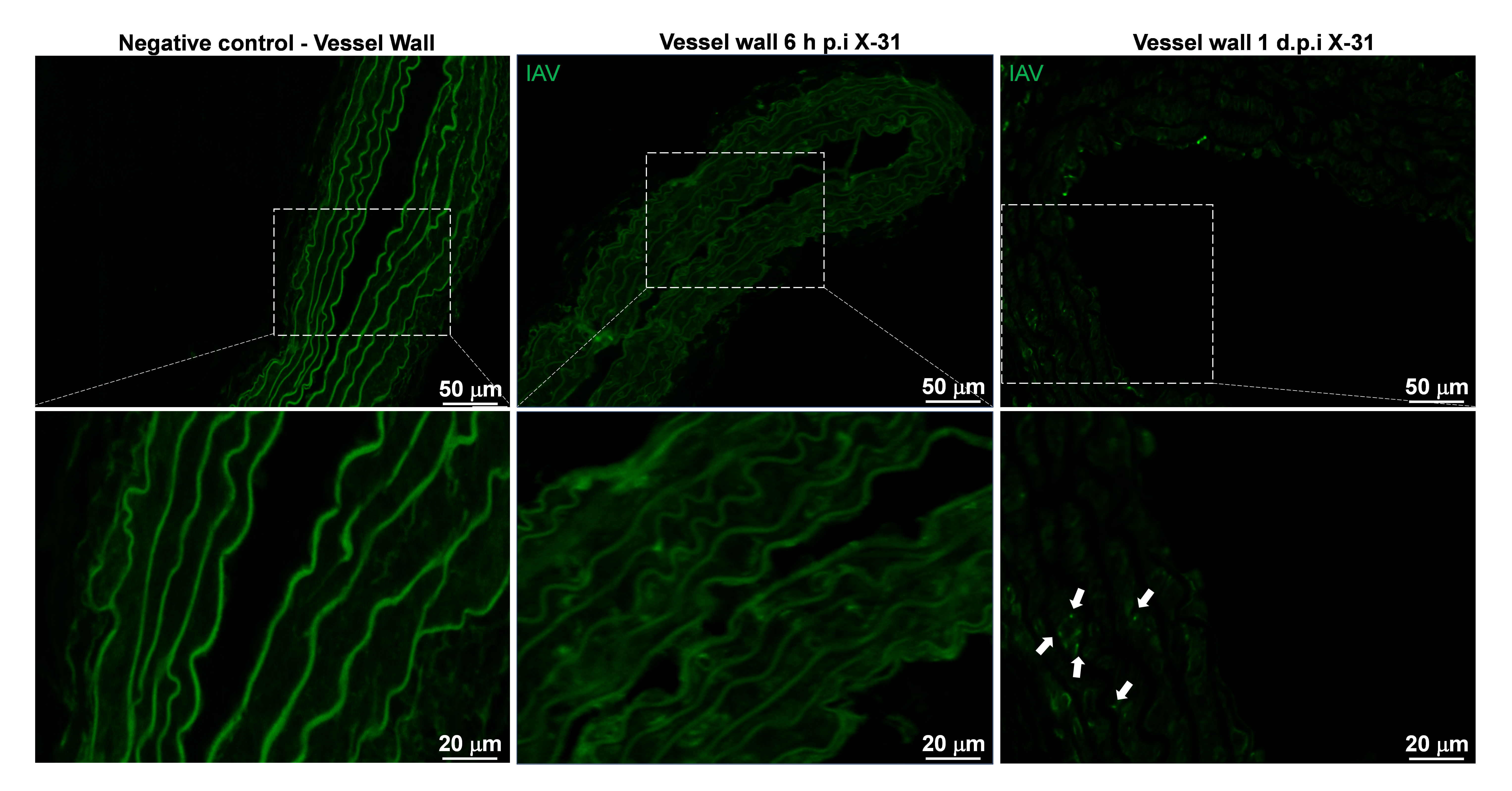

Supplement: S1 Fig — Representative immunofluorescence image of the arterial wall of pregnant Hk-x31 infected mice at 6 h and 1 d.p.i labeled with IAV nucleoprotein antibody (green). Negative control used to show level of autofluorescence. Data are representative of pregnant PBS, n = 5–6; pregnant X-31, n = 5–6; of at least two independent experiments. (TIF) [file ppat.1010703.s001.tif]

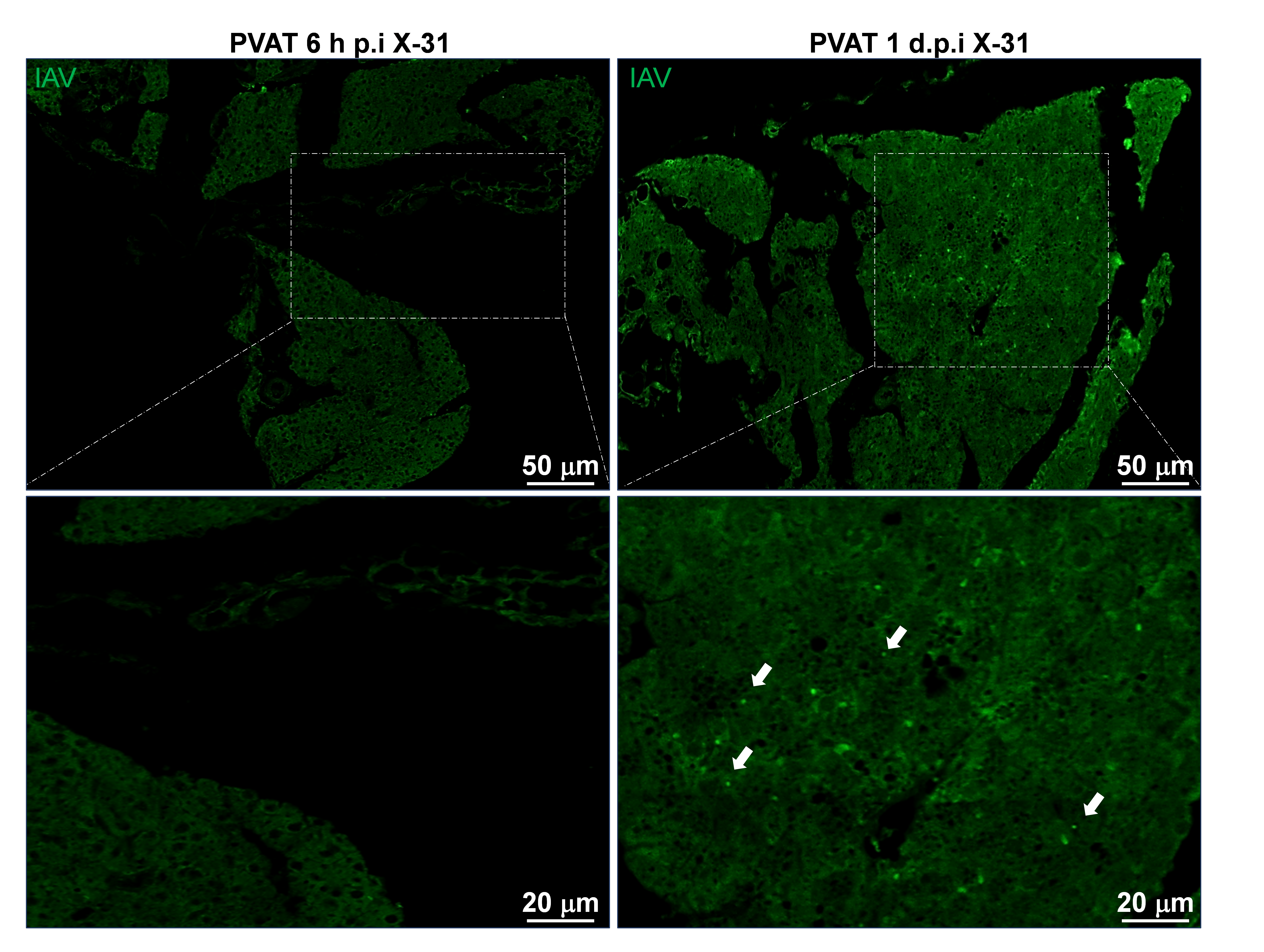

Supplement: S2 Fig — Representative immunofluorescence image of the PVAT of pregnant Hk-x31 infected mice at 6 h and 1 d.p.i labeled with IAV nucleoprotein antibody (green). Data are representative of pregnant PBS, n = 5–6; pregnant X-31, n = 5–6; of at least two independent experiments. (TIF) [file ppat.1010703.s002.tif]

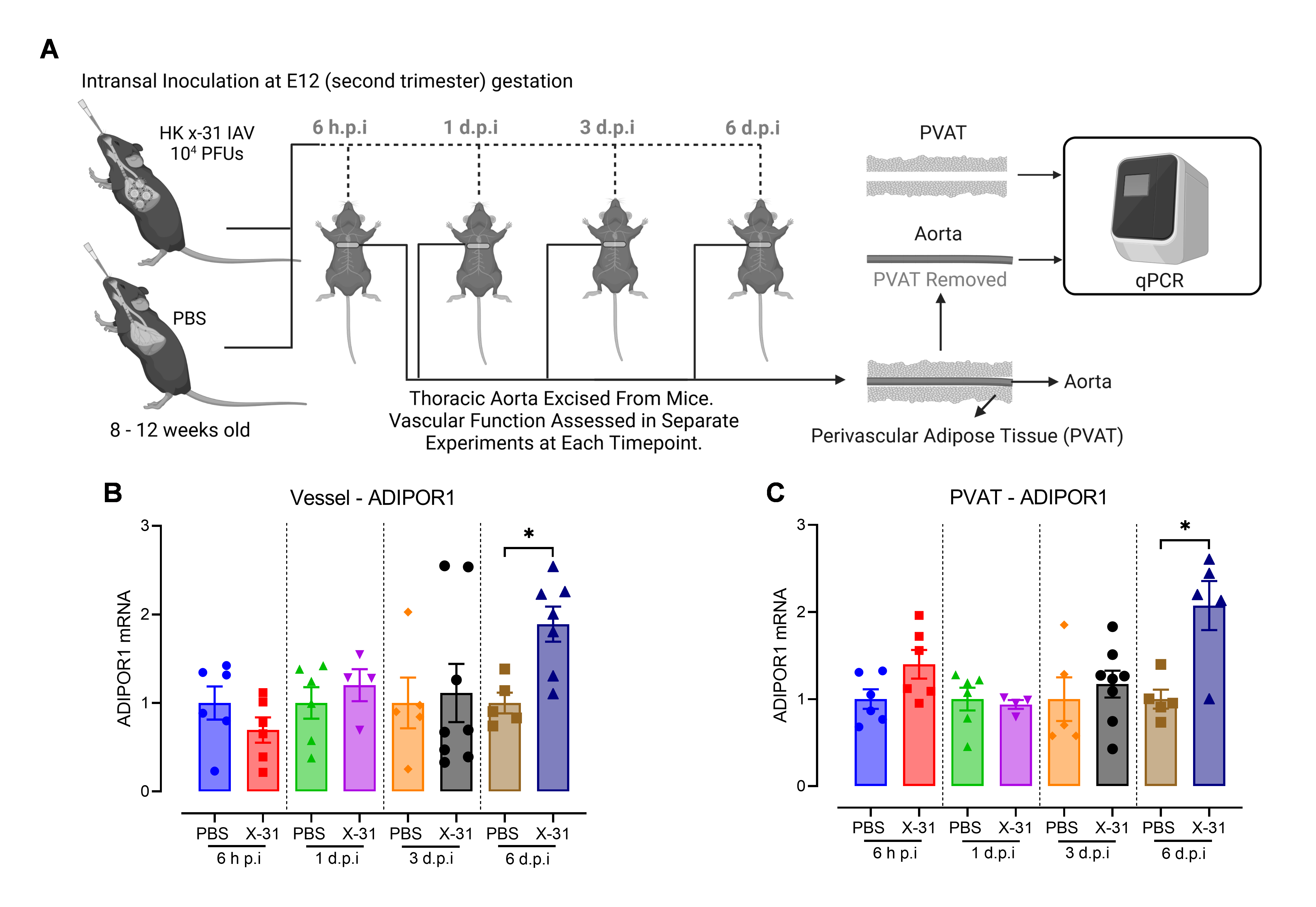

Supplement: S3 Fig — Pregnant mice were inoculated with PBS or Hk-x31 (X31; 104 PFU) for aortic assessment at 6 h, 1, 3 and 6 d.p.i. (A) Schematic of infection schedule and experiments (created with BioRender.com). (B) Adiponectin gene expression in the vessel wall of pregnant mice (C) Adiponectin gene expression in the PVAT of pregnant mice. Data are represented as mean ± SEM (pregnant PBS, n = 6–8; pregnant X-31, n = 6–8; of at least two to three independent experiments). All fold change calculations of the X-31 group were measured via qPCR, performed against the PBS group within its respective timepoint and normalised against RPS18. Statistical analysis was performed using unpaired t-test against the respective PBS control. * P<0.05. (TIF) [file ppat.1010703.s003.tif]

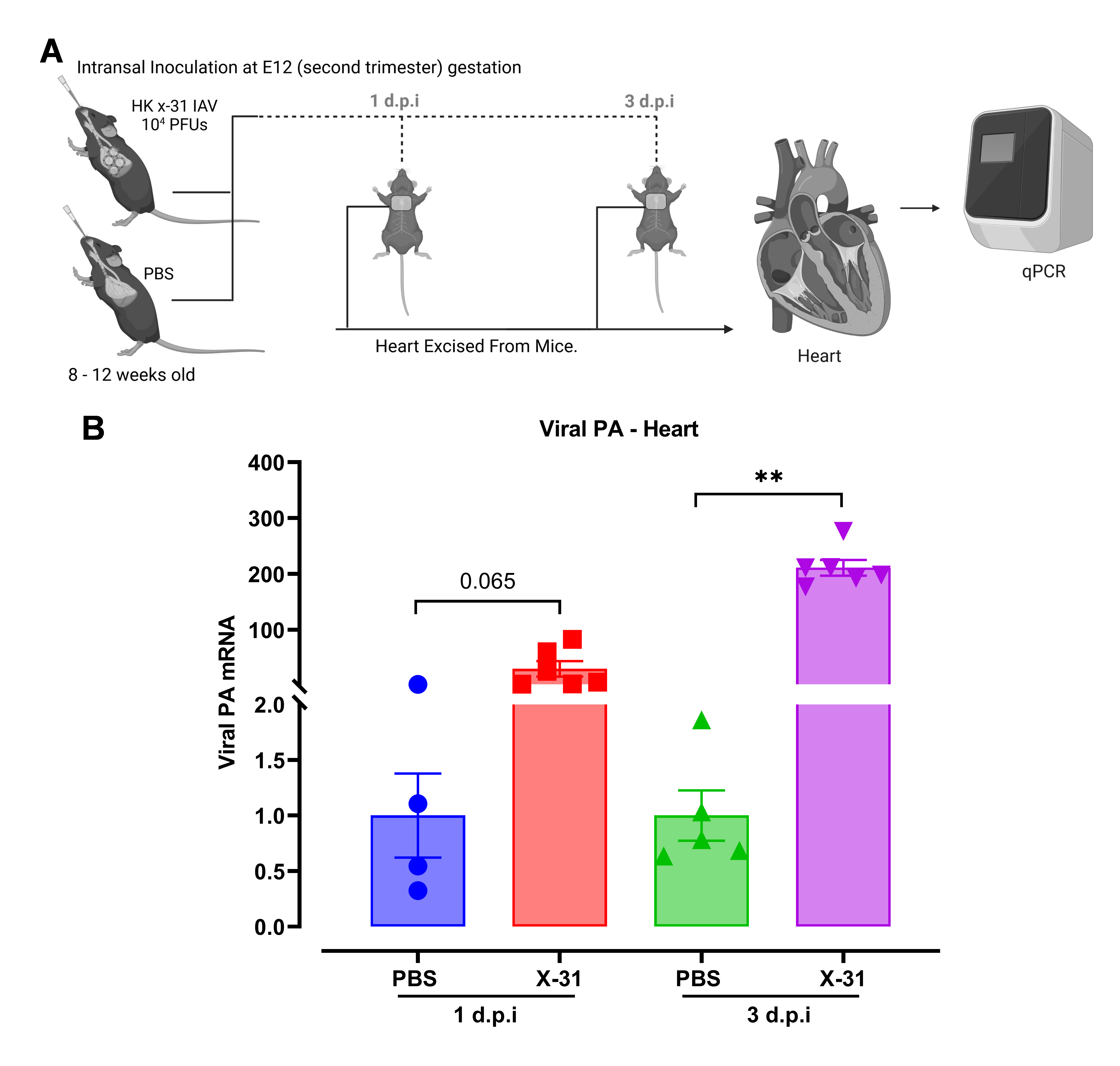

Supplement: S4 Fig — Pregnant mice were inoculated with PBS or Hk-x31 (X31; 104 PFU) for aortic assessment at 1 and 3 d.p.i. (A) Schematic of infection schedule and experiments (created with BioRender.com). (B) Viral PA gene expression in the heart of pregnant mice. Data are represented as mean ± SEM (pregnant PBS, n = 4–6; pregnant X-31, n = 4–8; of at least two to three independent experiments). All fold change calculations of the X-31 group were measured via qPCR, performed against the PBS group within its respective timepoint and normalised against RPS18. Statistical analysis was performed using unpaired t-test against the respective PBS control. * P<0.05, ** P<0.01. (TIF) [file ppat.1010703.s004.tif]

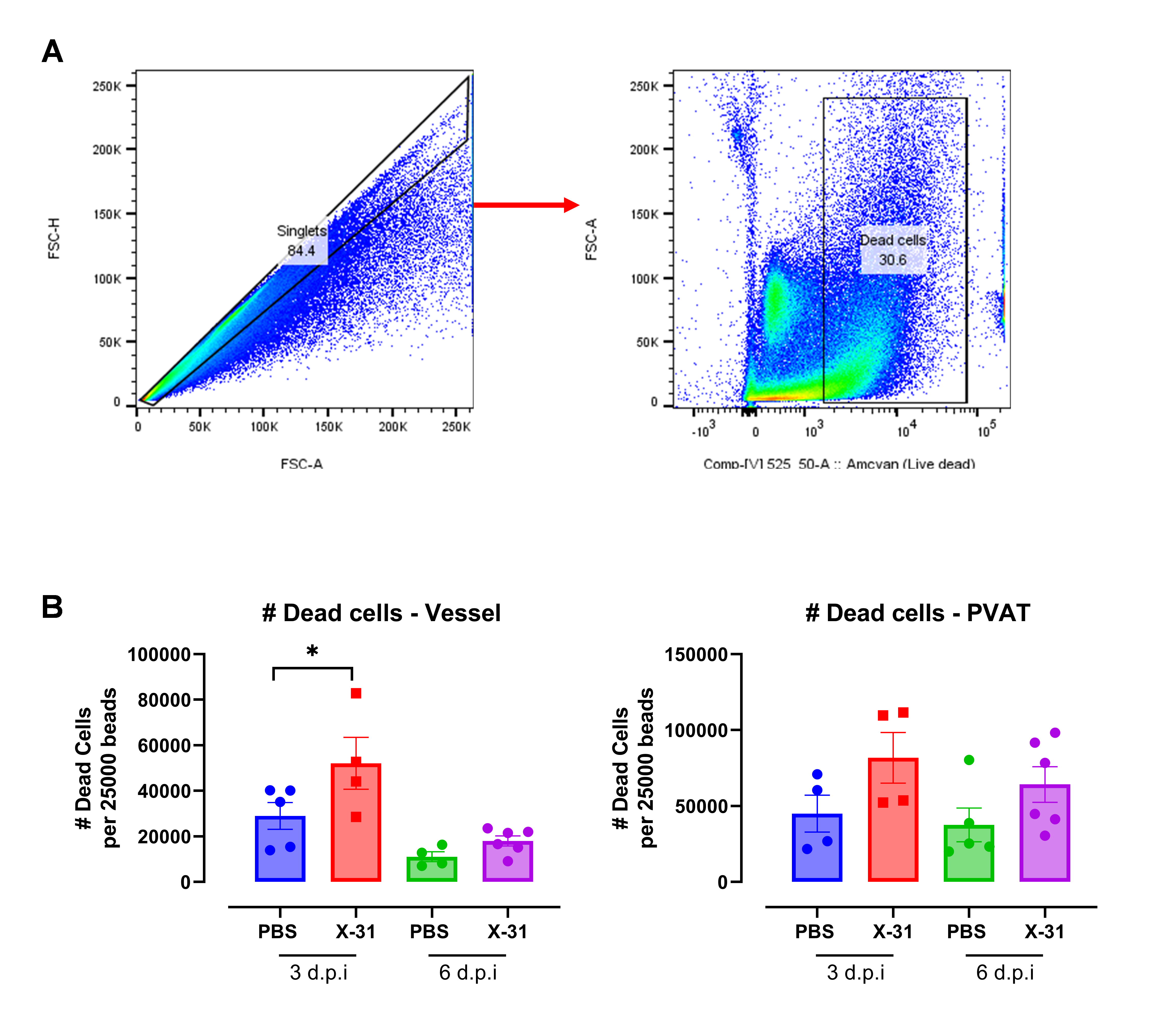

Supplement: S5 Fig — (A) Gating strategy for dead cells identification (B) Number of Dead cells in the vessel and PVAT per 25000 beads. Data are represented as mean ± SEM (pregnant PBS, n = 3–6; pregnant X-31, n = 4–6; of at least two independent experiments). Statistical analysis was performed using unpaired t-test against their respective PBS control. * P<0.05. (TIF) [file ppat.1010703.s005.tif]

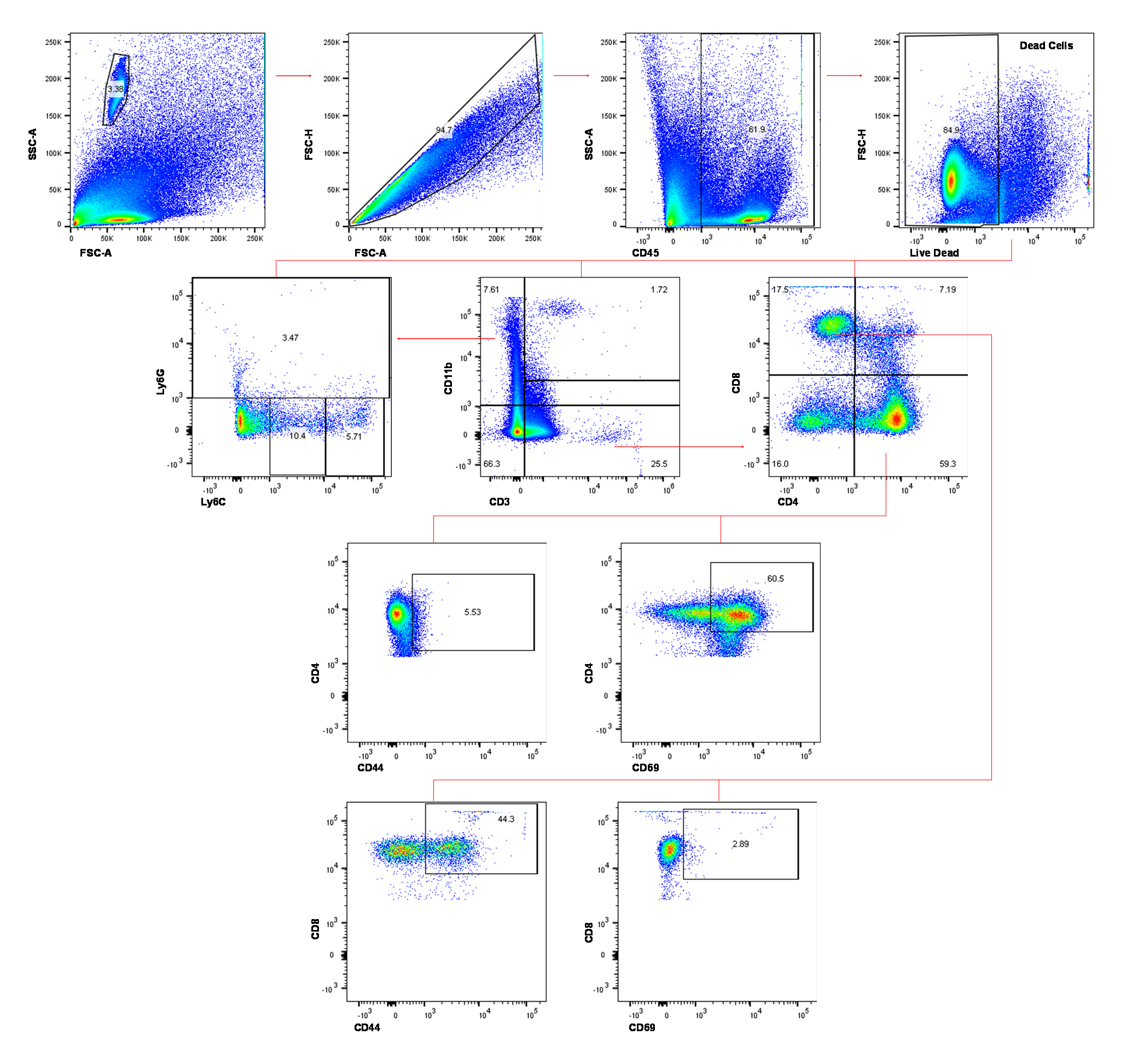

Supplement: S6 Fig — T cells and macrophages were gated as CD3+ and CD11b+ and respectively from CD45+ Lymphocytes. Patrolling Ly6Clow, pro-inflammatory Ly6Chigh monocytes and inflammatory Ly6G+ neutrophils were identified within CD11b+ macrophages. CD3+ T cells were further divided into subsets T helper (CD4+) and cytotoxic (CD8+) T cells. CD44+ and CD69+ were gated within CD4+ and CD8+ T cell subsets. (TIF) [file ppat.1010703.s006.tif]
