# Supplementary material for: Influenza A virus elicits peri-vascular adipose tissue inflammation and vascular dysfunction of the aorta in pregnant mice
Source: PLoS Pathog. 2022 Aug 5;18(8):e1010703. doi: 10.1371/journal.ppat.1010703 (PMC9385053; doi:10.1371/journal.ppat.1010703)
Supplement: S1 Table — Low Ct value represents higher infection and replication. (DOCX) [file ppat.1010703.s007.docx]

**Supplementary Table S1**

**Viral polymerase cycle threshold (Ct) values of vessels and perivascular adipose tissue from control and infected dams from 6 h, 1, 3 and 6 d.p.i study timepoints. Low Ct value represents higher infection and replication.**

| Study endpoint | Treatment group ^b^ | Aorta layer | Cycle threshold value ^a^ |
| --- | --- | --- | --- |
| 6 h.p.i | Pbs | Vessel | 32.19 |
|  | **Pbs** | **PVAT** | **32.17** |
|  | X-31 | Vessel | 31.47 |
|  | **X-31** | **PVAT** | **30.94** |
| 1 d.p.i | X-31 | Vessel | 25.33 |
|  | **X-31** | **PVAT** | **24.08** |
| 3 d.p.i | X-31 | Vessel | 27.37 |
|  | **X-31** | **PVAT** | **25.20** |
| 6 d.p.i | X-31 | Vessel | 27.74 |
|  | **X-31** | **PVAT** | **25.40** |

**^a^** Cycle threshold values for vessel wall and Peri Vascular Adipose Tissue (PVAT) control and infected dams obtained with RT-PCR

**^b^** *n = 4-6* in both Pbs controls and IAV infected groups
